# Phase Angle Association with Dietary Habits and Metabolic Syndrome in Diabetic Hypertensive Patients: A Cross-Sectional Study

**DOI:** 10.3390/nu14235058

**Published:** 2022-11-28

**Authors:** Dora Bučan Nenadić, Josipa Radić, Ela Kolak, Marijana Vučković, Ivana Novak, Marija Selak, Mislav Radić

**Affiliations:** 1 Nutrition and Dietetics Department, University Hospital of Split, 21000 Split, Croatia; 2 Department of Internal Medicine, School of Medicine, University of Split, 21000 Split, Croatia; 3 Internal Medicine Department, Nephrology and Haemodialysis Division, University Hospital of Split, 21000 Split, Croatia; 4 Internal Medicine Department, Rheumatology, Allergology, and Clinical Immunology Divison, University Hospital of Split, 21000 Split, Croatia

**Keywords:** phase angle, metabolic profile, diabetes, hypertension, dietary habits

## Abstract

Phase angle (PhA) levels are often lower than normal because both disease-specific parameters and disease-related inflammatory status, metabolic syndrome (MetS) included, can affect PhA. Therefore, the aim of this cross-sectional study was to compare body composition, metabolic profile and dietary patterns of participants with arterial hypertension (AH), type 2 diabetes mellitus (T2DM) and MetS with regard to PhA values. A total of 208 participants were included, of whom 53.6% were males. For each participant, data about body composition and anthropometric parameters, clinical and laboratory parameters, as well as food frequency questionnaire (FFQ) and Mediterranean Diet Serving Score (MDSS) were obtained. MC-780 Multi Frequency Segmental Body Mass Analyzer (Tanita) was used to assess body composition. Furthermore, waist-to-hip ratio (WHR) and waist-to-height ratio (WHtR) were calculated. The results showed that 75 (36.06%) participants had low PhA values and 133 (63.94%) had high PhA values. Participants with higher PhA values had significantly higher body fat percentage (*p* = 0.04), fat-free mass (kg; *p* < 0.001), muscle mass (kg; *p* < 0.001), skeletal muscle mass (% and kg; *p* < 0.001), sarcopenic index (SMI; *p* < 0.001) and mid-upper arm circumference (MUAC; *p* = 0.04), as well as lower fat mass percentage (*p* = 0.04). Regarding food frequency consumption, significantly higher intakes of red meat (*p* = 0.003), poultry (*p* = 0.02) and fast food (*p* = 0.003) were noticed in participants with higher PhA values. Adherence to the Mediterranean Diet (MeDi) was exceptionally low in both groups of participants, with significantly higher fish intake noticed in participants with high PhA (*p* = 0.03). In conclusion, our results showed that body composition could be the indicator of PhA in MetS as well as overall low adherence to the MeDi principles. These findings highlight the importance of adequate nutritional strategies and novel approaches to maintaining optimal body composition and adopting proper eating habits within the framework of one’s disease.

## 1. Introduction

Metabolic syndrome (MetS), also known as syndrome X, belongs to a group of non-communicable diseases that healthcare professionals and scientists have characterized as a set of predefined metabolic conditions such as hyperglycemia, dyslipidemia, arterial hypertension (AH) and central obesity [1,2].

Regufe et al. have shown that MetS is a series of disorders that, when associated with other pathologies, cause serious alterations in the human body that affect precisely patients with type 2 diabetes (T2DM) [3]. It is also noted that MetS is found in about one third of patients with AH, in whom the risk of cardiovascular (CV) and renal events increases significantly, even in the absence of overt diabetes [3].

The prevalence of MetS is closely related to socioeconomic factors as well as to lifestyle changes resulting from the impact of Western diet and sedentary lifestyle [1].

The etiology of MetS is very complex, being related to genetic mechanisms and environmental factors that predispose it, and mainly includes obesity associated with insulin resistance and secretion of inflammatory cytokines by adipose tissue that can cause damage and loss of cellular function [4,5,6].

Obesity, predominantly abdominal obesity, is one of the major factors contributing to the development and progression of MetS. Specifically, body fat mass used as a parameter for assessment of adipose tissue accumulation in the body has a central role in metabolic disorders [7]. Weight gain increases the amount of not only subcutaneous body fat but also intra-abdominal visceral fat, muscles, liver and beta cells, which results in insulin resistance, the appearance of MetS, and increased risk of developing both T2DM and CV diseases [8]. The enhanced subclinical inflammation characteristic of obesity also contributes to a high CV risk due to alterations in the metabolic profile such as dysglycemia or insulin resistance, elevation of triglycerides (TG) and reduction in high-density lipoprotein (HDL) cholesterol. Such a metabolic profile that can be noticed in very obese patients is also associated with changes in body volume status and reduced cellular integrity, which can be determined by phase-angle (PhA) measurement [9]. PhA is a good indicator of the body cell mass with which it is proportional but also reflects cellular integrity and function of cell membranes. It has been proposed as a prognostic parameter for changes in the metabolic profile and mortality in various chronic diseases [10], including cancer, and is associated with risk of morbidity in diabetes [11] and obesity [12].

PhA is a measure of cell stability estimated and interpreted by bioelectrical impedance analysis (BIA) and is a simple and rapid tool in the clinical setting [13].

It is calculated as the ratio between tissue resistance (R), which depends mainly on tissue hydration, and tissue reactance (Xc), which is related to cellularity, cell size and cellular integrity [14,15].

In disease conditions, including MetS, PhA levels are often lower than normal because both disease-specific parameters and disease-related inflammatory status can affect PhA [16]. Longo et al. have shown that low PhA became evident with the increase in waist circumference [17]. In addition, Dittmar et al. have shown that PhA is inversely correlated with glycated hemoglobin (HbA1C) in diabetic patients, being a marker of poor disease control [11]. Additionally, PhA reflected cell function and body cell mass (BCM) in T2DM patients [18]. As previous studies have shown diet is an indispensable component of therapy in the management of chronic diseases such as MetS and T2DM, but data on the relationship between diet and PhA in diabetic hypertensives are lacking.

De França et al. [19] have shown a significant positive association between the consumption of individual dietary components and PhA, such as extra virgin olive oil, cereals, legumes and meat in individuals older than 18 years. 

In a study by Barrea et al. conducted with healthy adults aged 18 to 59 years, a positive association between the Mediterranean diet (MeDi) and PhA independent of sex, age and body mass index (BMI) was found and they recommended nutritional screening as a good clinical practice when evaluating the PhA in the clinical setting [20].

Therefore, the aim of the present study was to assess the association between PhA and dietary habits, and the metabolic profile in Dalmatian patients diagnosed with T2DM, AH and MetS.

## 2. Materials and Methods

### 2.1. Study Design and Population

This cross-sectional study was carried out at the Outpatient Clinic for Clinical Nutrition, Nephrology and Haemodialysis Division, Internal Medicine Department, University Hospital of Split, Croatia, between March 2019 and April 2020. Out of six hundred and twenty-four patients recruited during their regular visit to the nephrologist and dietitian, two hundred and eight of them had diagnosed both AH and T2DM as well as defined MetS. To define MetS, the criteria from the International Diabetes Foundation (IDF), which relies mainly on central obesity, were applied. MetS is present if three or more of the following markers are met:

- Central adiposity defined as waist circumference (WC) ≥ 94 cm for European males and ≥ 80 cm for European females;
- Fasting blood glucose (FBG) ≥ 5.6 mmol/L or diagnosed diabetes;
- HDL cholesterol < 40 mg/dL (males) and < 50 mg/dL (females) or treatment for low HDL concentration;
- Serum TG > 150 mg/dL or treatment for hypertriglyceridemia;
- Blood pressure (BP) > 130/85 mmHg or treatment for hypertension.

**Figure 1:**
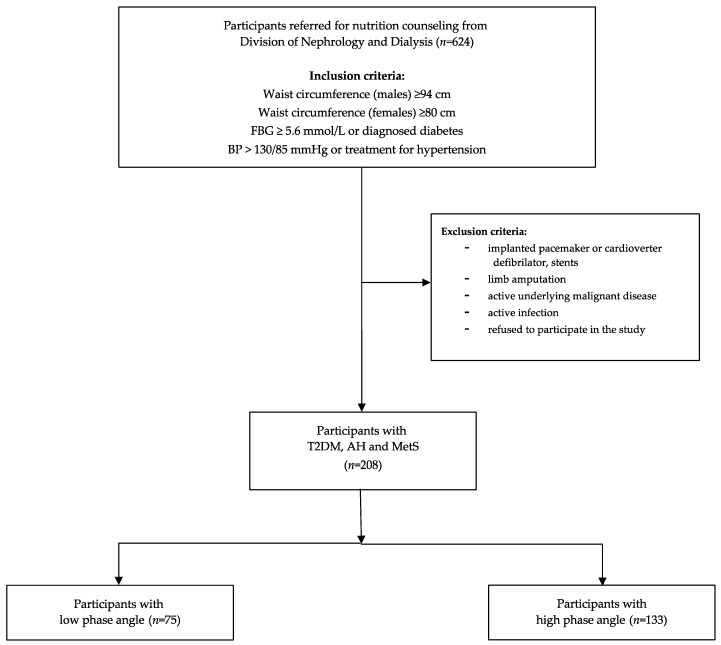
Study design. Abbreviations: FBG—fasting blood glucose; BP—blood pressure; T2DM—type 2 diabetes mellitus; AH—arterial hypertension; MetS—metabolic syndrome.

Patients who met one of the following criteria were excluded from the study: had implanted pacemaker or cardioverter defibrillator, stents, or limb amputation; had an active underlying malignant disease or active infection; and refused to participate in the study. The study flowchart is shown in Figure 1.

All participants were informed about the purpose of the study and written as well as oral consent was obtained for each participant.

The study was conducted following the guidelines of the latest version of the declaration of Helsinki, and its protocol was accepted by the Ethics Committee of the University Hospital of Split on 29 March 2019 (Ur.no. 2181-147-01/06/M.S.-19-2, Class: 500-03/19-01/20.)

### 2.2. Body Composition, Anthropometric and Blood Pressure, and Arterial Stiffness Measurement

Using an MC-780 Multi Frequency Segmental Body Mass Analyzer (Tanita, Tokio, Japan), body composition was assessed for each study participant. The scale is based on a technology called bioelectrical impedance analysis (BIA). BIA provides data regarding body mass (kg), muscle mass (kg and %), fat-free mass (kg and %), visceral fat, trunk fat mass (kg and %), skeletal muscle mass (kg and %), sarcopenic muscle index (SMI), PhA (°), total body water (TBW, kg), extracellular water (EW, kg) and intracellular water (IW, kg). Resistance (R, Ω) and reactance (Xc, Ω) were estimated and PhA was calculated according to the following equation: arctangent Xc/R ((Xc/R) x (180/π) [10]. The patients were instructed not to take any food or liquid at least 3 h before the measurement; to urinate just before the measurement; and not to consume alcohol, eat or drink excessively or to exercise excessively at least a day before the body composition measurement according to device manual specifications [21].

To measure the circumference of the mid-upper arm (MUAC), hip (HC) and WC, non-stretchable, flexible body measuring tape was used. The MUAC is defined as relaxed, with the body stretched by the hand, with a measuring tape placed horizontally 1 cm above the middle of the upper arm. The WC is defined above the navel in the standing position facing forward of the examinee, with the measuring tape set horizontally. HC is defined around the widest portion of the buttocks, with the tape parallel to the floor. Height was measured using a stadiometer. For each study participant BMI, as well as WHR and WHtR were calculated.

Peripheral and central BP and arterial stiffness measurements were performed using a Agedio B900 (IEM, Stolberg, Germany) device based on the principle of oscillometry. The right-sized cuff was selected according to the upper arm circumference and positioned accurately. All participants were measured in a relaxing environment, comfortably seated with their back and arm supported, feet flat on the ground, legs not crossed and with an empty bladder. Data about peripheral systolic blood pressure (pSBP), peripheral diastolic blood pressure (pDBP) and Pulse Wave Velocity (PWV; m/s) were obtained.

### 2.3. Lifestyle Questionnaire and Mediterranean Diet Serving Score

The lifestyle questionnaire, consisting of a series of questions on sociodemographic information, dietary and smoking habits, was administered by a qualified dietitian. Additionally, several questions from the NHANES (National Health and Nutrition Examination Survey) were included to form a concise, self-administered dietary survey for assessment of the frequency of intake in the last 6 months. Our food frequency questionnaire consisted of 23 questions divided into 5 groups (proteins, dairy, fruits and vegetables, grains, and sweets and crisps). Mediterranean Diet Serving Score (MDSS) was administered to evaluate adherence to the MeDi principles according to the recommended frequency of consumption of fourteen (14) different food items as well as food groups (MeDi components). According to Monteagudo et al., this semiquantitative food frequency questionnaire is considered a validated, easily applicable and accurate tool for estimating adherence to the MeDi [22]. Based on a MeDi food pyramid, three points were assigned for the recommended intake of food if consumed with every meal (cereals, olive oil, vegetables, and fruit). Next, two points were scored for the daily consumption of dairy products and nuts, and finally, one point was assigned for the recommended weekly intake of potatoes (≤3), legumes (≥2), eggs (2–4), poultry (2), red meat (<2), fish (≥2), sweets (≤2) and fermented beverages (1 and 2 glasses a day for females and males, respectively) [23]. A total value of zero was given for the intake higher or lower than the recommendations for any MeDi component. According to the original study, the MDSS ranges from zero to twenty-four points with the optimal cut-off point set at ≥13.5 to determine the adherence to the MeDi principles [22].

### 2.4. Medical History and Clinical and Laboratory Parameters

Data on the length of treatment for T2DM and AH as well as the other co-existing diseases, such as chronic kidney disease (CKD), and medical therapy were obtained for each study participant during clinical examination and from their medical records.

All study participants underwent usual peripheral blood sampling on the same day of the body composition and BP measuring. The collected data included the following laboratory parameters: serum albumin (g/L), glucose (mmol/L), HbA1c (%), uric acid (mmol/L), TG (mmol/L), total cholesterol (mmol/L), low-density lipoprotein cholesterol (LDL; mmol/L), HDL cholesterol (mmol/L); urea (mmol/L), creatinine (mmol/L), uric acid (mmol/L), mean cellular volume (MCV; fL), potassium (mmol/L), phosphates (mmol/L), calcium (mmol/L), estimated glomerular filtration rate (eGFR) using CKD-EPI (mL/min/1.73 m^2^), 24 h (measured) albuminuria (mg/g), proteinuria (mg/g) and albumin-to-creatinine ratio (ACR; mg/g).

Blood samples for analysis of serum levels of complement components were collected in standard test tubes without additives in our Laboratory of Medical Diagnostics and Biochemistry at the University Hospital of Split, Croatia, and 30 min later, were centrifuged for 10 min at 1690× *g* on HERMLE Z400 centrifuge model (Hermle Labortechnik GmbH, Wehingen, Germany). For creatinine measurement, the Jaffe method was used. A complete blood count was obtained using a hematology analyzer (Advia 120, Siemens, Erlangen, Germany).

### 2.5. Statistical Analysis

Categorical data were presented by absolute and relative frequencies. The normality of the distribution of continuous variables was tested by the Shapiro–Wilk test. Continuous data were described by the median and the limits of the interquartile range (IQR). The Mann–Whitney U test was used to compare the median between two groups, while the Chi-squared test was used to analyze the differences between proportions. Logistic regression analysis (multivariate—stepwise method) was used to analyze the independent factors associated with development of high PhA. The variable PhA was categorized into terciles; its values were classified by tercile distribution (1st tercile < 5.2; 2nd tercile 5.2 to 6.0; 3rd tercile ≥ 6.0). This variable was dichotomized for the analysis (0 = low PhA (1st tercile), and 1 = high PhA (2nd and 3rd terciles)). All *p*-values were two-sided. The level of significance was set at an alpha of 0.05. The statistical analysis was performed using MedCalc^®^ Statistical Software version 20.100 (MedCalc Software Ltd., Ostend, Belgium; https://www.medcalc.org; accessed on 24 July 2022) and IBM SPSS Stat. 23 (IBM Corp. Released 2015. IBM SPSS Statistics for Windows, Version 23.0. IBM Corp: Armonk, NY, USA.).

## 3. Results

The sample comprised 208 adult hypertensive diabetic patients with a defined MetS, where most of the participants were males (56.3%) and obese (54.8%). Data about body composition and anthropometric parameters, as well as clinical parameters of all study participants, including differences regarding PhA categories, are shown in Table 1.

Out of all study participants, 75 (36.06%) of them had low PhA values and 133 (63.94%) had a high PhA values. Male participants had significantly higher PhA (*p* < 0.001) while older participants had significantly lower PhA (*p* < 0.001). Among participants in the low PhA category, the prevalence of overweight was 33.33% and the prevalence of obesity was 52%. Among those participants with high PhA, 36.84% were overweight and 56.39% were obese without significant difference between two groups of participants. Given the body composition and anthropometric measurements, significantly higher fat-free mass (kg; *p* < 0.001), muscle mass (kg; *p* < 0.001), skeletal muscle mass (% and kg; *p* < 0.001), SMI (*p* < 0.001) and MUAC (*p* = 0.04) were observed in participants with high PhA. Also, a significantly lower fat mass percentage (*p* = 0.04) was noticed in the same group of participants. Regarding clinical parameters, participants with low PhA had significantly higher PWV values (*p* = 0.006) and higher intake of oral antidiabetics (*p* = 0.003) whereas participants with high PhA had significantly higher pDBP (*p* = 0.04), insulin (*p* = 0.009), and angiotensin-converting enzyme (ACE) inhibitors intake (*p* = 0.04).

Data about biochemical parameters of all study participants with differences regarding PhA categories are shown in Table 2. Statistically significant differences were noticed for eGFR (*p* = 0.02), erythrocytes (*p* = 0.04) and hemoglobin (*p* < 0.001) with higher values observed in high PhA category.

Dietary habits of the total study population including differences regarding PhA are shown in Table S1; 52.4% (109) of participants had received dietary recommendations regarding their comorbidities, while 44.9% (49) of them did not adhere to the recommendations without significant difference between PhA categories. Most of the study population (77.9%) have between 2 and 4 meals per day; meanwhile, 14.4% of the participants skip breakfast. Regarding food frequency consumption, a significantly higher intake of red meat (*p* = 0.003), poultry (*p* = 0.02) and fast food (*p* = 0.003) was noticed in participants with higher PhA values.

**Figure 2:**
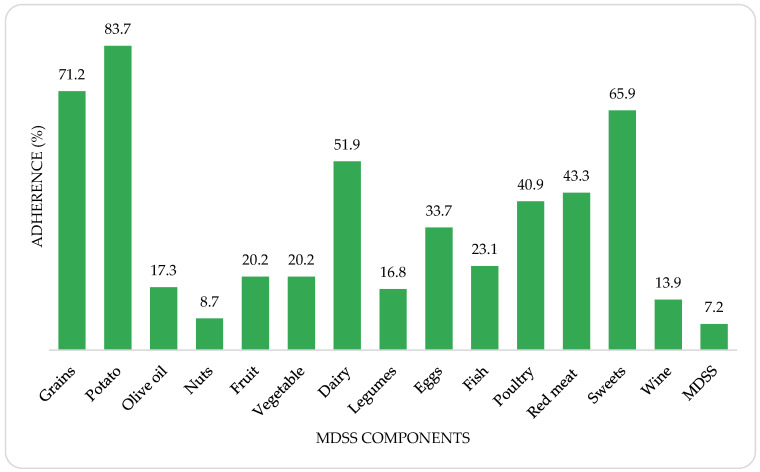
Adherence to the MeDi and its components among all study population.

Adherence to the MeDi and its components among all study population is shown in Figure 2. Low adherence to the principles of the MeDi was noticed in all study participants, where only 15 (7.2%) MetS participants scored 14 or more points on the total MDSS score. The highest adherence for individual MeDi components was found for potatoes (83.7%), grains (71.2%) and sweets (65.9%), whereas the lowest adherence was determined for nuts (8.7%), wine (13.9%) and legumes (16.8%) intake.

**Figure 3:**
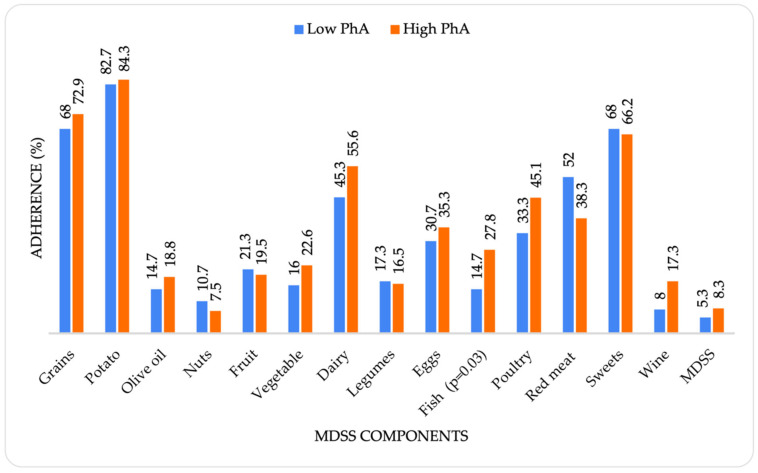
Adherence to the MeDi considering phase angle values.

When observing the MeDi adherence considering PhA values, as shown in Figure 3, no significant difference was found for the total MeDi score. Exceptionally low adherence to the MeDi principles was noticed in both PhA categories, with only 5.3% (4) of participants with low PhA values and 8.3% (11) of participants with high PhA values adhering to the MeDi recommendations. Regarding each MeDi component, a significant difference was determined only for fish intake (*p* = 0.03), with higher adherence to the specific recommendation in participants with high PhA values.

Correlations between PhA and measured parameters are shown in Table 3 (only statistically significant correlations are shown). Significant positive correlations were found for WC (*p* = 0.004); pDBP (*p* = 0.01); fat-free mass (*p* < 0.001); muscle mass (kg; *p* = 0.01 and %; *p* < 0.001); skeletal muscle mass (kg; *p* < 0.001 and %; *p* < 0.001); SMI (*p* < 0.001); erythrocytes (*p* = 0.02); hemoglobin levels (*p* < 0.001); eGFR (*p* = 0.02); HbA1c (*p* = 0.04); and frequency of deli meats (*p* < 0.001), eggs (*p* = 0.03) and cheese intake (*p* = 0.03). Significant negative correlations were found for age (*p* < 0.001), PWV (*p* = 0.04), metabolic age (*p* = 0.01), body fat percentage (*p* = 0.01), frequency of fruit intake (*p* = 0.03) and MeDi adherence to red meat intake (*p* = 0.04).

Associations between PhA and measured parameters adjusted for age, gender and BMI are shown in Table 4. A significant association was found between MUAC, skeletal muscle mass (kg), hemoglobin levels and HbA1c.

## 4. Discussion

To our knowledge, this is the first study that evaluated PhA values in patients with T2DM, AH and MetS. Because PhA is used as a marker of inflammatory diseases, including obesity, its value is usually lower than normal and could be increased with improvement of clinical status [24]. One of the recommended strategies to achieve this improvement is a change in lifestyle, mainly by following the principles of the MeDi [25,26]. Therefore, the main objective of our study was to determine association between PhA and clinical status as well as adherence to the MeDi in this specific patient population.

The present study included 208 hypertensive diabetic participants with MetS defined according IDF criteria [27], with 133 of them in a high PhA category.

One of the most important determinants of PhA in healthy population are age, sex and BMI with higher PhA values noticed in younger subjects and males due to the differences in body composition that is lower fat percentage and higher muscle mass [28], which is in line to findings in our study. PhA is increasing with an increase in BMI but it is inversely associated with BMI value higher than 40 kg/m^2^ [15,29]. Furthermore, a decrease in muscle mass is associated with a decrease in PhA values [30], which is in line with the results presented in this study. Similarly to our results, Jaremkow et al. reported that PhA values positively correlated with fat-free mass, muscle mass and muscle percentage, whereas negative correlations were determined for fat mass and fat percentage [31]. On the other hand, Curvello-Silva found no correlation between PhA and body composition parameters in severely obese patients [9]. Gonzalez et al. showed that fat-free mass is the most significant predictor of PhA after age [32]. In the study on 85 healthy adults, predictors of PhA were muscle mass and visceral fat [33]. Contrary, in a study conducted with 1967 healthy adults, PhA increased with an increase in BMI and was inversely associated with fat percentage but only in men [14]. Furthermore, fat percentage was another predictor of PhA along with sex, age and BMI [14]. Correlations between PhA and body composition parameters as well as anthropometric parameters were also confirmed by the predictive analysis in this study.

The results from our study showed that significantly higher PWV value, as an indirect marker of arterial stiffness, was found in a low PhA group. This finding is in line with previous studies. A recent study found negative correlation between PhA and intima-media thickness, another parameter of arterial stiffness, in kidney transplant recipients [34], which was similar to findings in our population. In addition, a Danish study found an association between lower PhA value and higher incidence of CV disease over a 24-year period, even after adjustment for potential confounders, especially in women [35].

Our results also show that participants with AH, T2DM, MetS and high PhA values were almost twice as likely to use insulin than those with low PhA values, whereas the duration of T2DM treatment was not significantly different between the groups. The relationship between insulin and weight gain observed through adipose tissue increase in both T2DM and T1DM is already established. It results from an unconscious calorie increase due to the fear of hypoglycemia or acquired control over blood glucose levels, a reduction in glycosuria, as well as central effects on weight and appetite regulation [36,37].

Therefore, significant differences in HbA1c levels or FBG were not found between these two groups of patients. The type of insulin and daily number of injected units was not taken into consideration when conducting present study, and it is a limitation that could represent a bias in interpreting our results. Average HbA1c of 6.8% (IQR 6.3–7.7) and average FBG being 4.7 mmol/L (IQR 4.1–5.75) were reported in the present study. The reason for the somewhat lower FBG levels observed in our participants may be the high prevalence of CKD, which simultaneously affects the reduction in gluconeogenesis and clearance of both endogenous and exogenous insulin [38,39]. After adjustment for age, BMI and gender, HbA1c remained as a significant positive predictor of PhA in our study. In contrast, Dittmar et al. reported that PhA has an inverse correlation with glycemic control [11]. The recent study on Korean T2DM patients also indicated that HbA1c and FBG were independently associated with PhA [18]. The differences in the results can be explained by the increased proportion of adipose tissue in participants with a higher PhA, which leads to worsening in a glycemic regulation due to an increased insulin resistance. In support of the above, significantly higher use of insulin was reported in the high PhA participants the present study, as well as non-compliance with dietary recommendations and principles of the MeDi, which is why the HbA1c levels were expectedly higher in this group of participants.

Additionally, participants with higher PhA had a significantly higher eGFR, which was expected considering that patients with better kidney function have fewer dietary restrictions. Furthermore, the relationship between malnutrition and progression of CKD is well known. Metabolic acidosis, hormonal dysregulation and systematic inflammation are a common occurrence among CKD patients and, as such, are attributed to the risk for negative nitrogen balance and, therefore, malnutrition [40]. Additionally, higher erythrocyte and hemoglobin levels might indicate a better nutritional status in this group of participants considering that higher eGFR levels are reciprocally associated with erythropoietin synthesis [41].

Of all study participants, 52.4% (109) had received dietary recommendations regarding their comorbidities, while 44.9% (49) of them did not adhere to the received recommendations without significant difference between PhA categories. Similar results were found in recent studies with T2DM patients and patients with cardiometabolic diseases [42,43]. Available data suggest that diabetic patients often find it problematic introducing changes in their lifestyle, as the most difficult part of the treatment [44].

Contrary to our results, Barrea et al. reported association between adherence to the MeDi and PhA independently of age, sex and body weight [20]. The discrepancies in the results could be explained by the questionnaire used to assess adherence to the MeDi. In this study, the MDSS questionnaire was used, which divides the results into two categories according to whether the principles of the MeDi are adhered to, in contrast to the aforementioned study, which used the PREvencion con DIeta MEDiterranea (PREDIMED) questionnaire, which has three categories. Additionally, considering that only 15 participants with MetS scored 14 or more points on the total MDSS score in our study, further statistical analysis would not be significant.

The highest adherence for individual MeDi components was found for potatoes, grains and sweets, whereas the lowest adherence was determined for nuts, wine and legumes intake. Considering that the study population were predominantly elderly people who are most affected by food insecurity [45], this finding is not surprising. Food insecurity reflects a lower quality in dietary intake and is one of the risks for developing several chronic diseases such as AH and T2DM [46]. Potatoes and grains, especially bread, which are traditionally consumed with every meal in our country, are considered readily available and inexpensive foods. A similar reason, high price was found for low nuts consumption. The high adherence to the recommendations for the sweets consumption was actually to be expected, as the consumption of sweets is usually associated with high blood glucose levels [47]. One of the main reasons for low compliance with the recommendations on wine consumption could be the high medication burden in these patients, which is explained by the interaction of medications and alcohol [48].

Regarding the individual MeDi components, a significant difference was found only for fish consumption, with participants with high PhA values more likely to follow this specific recommendation. Considering that PhA reflects muscle quality [28], a recent study examining the actual benefits of fish consumption in terms of the beneficial effects of the biological compounds contained in fish on muscle mass found that fish consumption has a protective and anti-inflammatory effect on skeletal muscle mass [49]. A previous study also demonstrated that the consumption of fish twice weekly over a 10-week period resulted in a significant increase in skeletal muscle mass and appendicular lean mass [50].

According to the FFQ, participants with higher PhA values were found to have a significantly higher frequency of red meat, poultry and fast-food consumption. In addition, positive correlations were found for PhA and frequency of deli meats, eggs, and cheese intake, as well as negative correlations for frequency of fruit intake and adherence to red meat intake. The characteristic of all of the above foods, with the exception of fruit, is a high protein content, which provides the body with energy and has a positive effect on PhA [51]. Similar results were reported by Jaremkow et al., who also confirmed a positive correlation between meat consumption and PhA in young adults. The correlation between the consumption of fast food and the PhA was also statistically significant, which may be due to the type of fast food consumed in our country, mainly traditional sausages, burgers and roasted chicken, which favors the consumption of meat [31].

In contrast, a high consumption of fruit could have negative effect on body composition, as the high fructose content has an adipogenic property [52]. Moreover, with a higher fruit intake and, therefore, of simple sugars, there is also a need to correct the insulin dosage accordingly, which may result in an increase in adipose tissue already mentioned [36].

Our study has some limitations, mainly due to its cross-sectional design, which as such does not allow for causal associations. In addition, it was conducted in a single tertiary center where a relatively large number of participants gravitated. A relatively small number of participants were included, but the sample was representative because it included a particularly vulnerable population suffering from T2DM, AH and MetS. Considering that included participants lived in the same geographical area and, therefore, had similar dietary pattern homogeneity of the sample could increase the selection bias. Potential bias in determining MeDi adherence and FFQ could be caused by questionnaire self-administration and unreliable responses regarding food intake recall and over-or underestimation of food intake, although a qualified dietitian was available to answer any interfering questions. There is a lack of data on medication dosage.

## 5. Conclusions

The obtained results indicate a high rate of overweight and obesity in subjects suffering from AH, T2DM and MetS without significant differences in PhA values. Furthermore, significantly higher values of fat-free mass, muscle mass, trunk muscle mass and lower values of body fat were noticed in participants with high PhA. It was determined that adherence to the principles of the MeDi was extremely low in both groups of participants regarding PhA values, while fish intake was significantly higher in participants with high PhA values. Considering that PhA is associated with muscle mass and inversely associated with fat mass, body composition could be useful indicator of PhA in patients with MetS.

Lifestyle changes, primarily through proper nutrition and regular age-appropriate physical activity, are of great importance in the treatment of chronic diseases including obesity. The MeDi is emerging as a dietary pattern that provides many health benefits in the aforementioned diseases, which is why the data obtained are extremely devastating. The MeDi can have favorable effects on the prevention and treatment of obesity, which is one of the major risk factors for HA, T2DM and MetS onset. These findings highlight the importance of adequate nutritional strategies to help patients regain optimal body composition as well as to understand their dietary regimens and to improve adherence to the received recommendations.

## Figures and Tables

**Table 1:** Basic characteristics of the study population including differences regarding phase-angle categories.

	Total (*n* = 208)	Low Phase Angle (*n* = 75)	High Phase Angle (*n* = 133)	*p*-Value *
Age (years), median (IQR)	68 (60–73)	72 (65–76)	65 (57–71)	<0.001
Sex, M/F	117/91	28/47	89/44	<0.001
Duration of DM treatment (years), median (IQR)	10 (4.75–20)	10 (5.75–20)	10 (3–20)	0.84
Duration of AH treatment (years), median (IQR)	10 (5–20)	15 (6–20)	10 (5–20)	0.27
Smoking, N (%)	43 (20.7)	15 (20)	28 (21.1)	0.74
pSBP (mmHg), median (IQR)	146 (134–160.5)	149.5 (131.5–162)	144 (134–160)	0.41
pDBP (mmHg), median (IQR)	91 (80–100)	89.5 (74–96.5)	93 (82.5–101.5)	0.04
PWV (m/s), median (IQR)	10.45 (9–11.8)	11.2 (10.1–12.05)	10 (8.68–11.43)	0.006
Pharmacological therapy				
BBs, N (%)	97 (46.6)	38 (52.1)	59 (46.8)	0.48
ACE inhibitors, N (%)	106 (51)	32 (43.8)	74 (58.7)	0.04
ARBs, N (%)	31 (14.9)	14 (19.2)	17 (13.6)	0.30
Cas, N (%)	114 (54.8)	41 (56.2)	73 (57.9)	0.81
Diuretics, N (%)	137 (65.9)	49 (67.1)	88 (69.8)	0.69
Moxonidine, N (%)	52 (25)	15 (20.5)	37 (29.4)	0.17
Urapidil, N (%)	17 (8.2)	6 (8.2)	11 (8.8)	0.89
Oral antidiabetics, N (%)	148 (71.2)	63 (86.3)	85 (67.5)	0.003
Insulin, N (%)	51 (24.5)	11 (15.1)	40 (31.7)	0.009
Statin, N (%)	101 (48.6)	37 (50.7)	64 (50.8)	0.99
Body composition				
Body fat (%), median (IQR)	29.05 (23.6–36.55)	30.5 (24.6–38.3)	28 (22.95–34.65)	0.04
Body fat (kg), median (IQR)	27.45 (20.18–35.85)	29.22 (19.4–35.4)	25.8 (20.25–36.8)	0.55
Fat free mass (kg), median (IQR)	65.55 (58.15–74.6)	59.7 (53.7–68.7)	67.7 (60.6–77.15)	<0.001
Trunk fat mass (kg), median (IQR)	14.3 (10.13–18.5)	14.1 (10.1–18)	14.5 (10.15–18.8)	0.60
Trunk fat mass (%), median (IQR)	28.3 (22.5–33.6)	28.4 (23.2–34)	28.2 (22–32.55)	0.43
Muscle mass (%), median (IQR)	67.5 (59.75–72.98)	66 (58.6–71.7)	68.3 (61.45–73.7)	0.06
Muscle mass (kg), median (IQR)	62.25 (55.25–70.75)	56.9 (51–65.3)	64.3 (57.55–73.3)	<0.001
Skeletal muscle mass (%), median (IQR)	37.05 (29.98–41.48)	33.1 (26.7–37.9)	38.6 (34.3–42.5)	<0.001
Skeletal muscle mass (kg), median (IQR)	34.2 (26.8–39.8)	27.7 (23.5–35.2)	36.8 (31.1–42.35)	<0.001
SMI, median (IQR)	9.08 (8.1–10.14)	8.4 (7.85–9.45)	9.28 (8.47–10.44)	<0.001
Metabolic age (years), median (IQR)	61 (54.25–66.75)	62 (56–69)	59 (53–65.5)	0.02
Anthropometric parameters				
WHtR, median (IQR)	0.63 (0.59–0.69)	0.64 (0.58–0.69)	0.63 (0.59–0.7)	0.61
WHR, median (IQR)	0.96 (0.91–1.02)	0.94 (0.9–1.01)	0.98 (0.93–1.02)	0.16
MUAC (cm), median (IQR)	32.55 (29.6–35.5)	32 (29.5–34)	33 (30–36)	0.04
WC (cm), median (IQR)	111 (103–120)	110 (101–119)	111.7 (104–120.5)	0.08
BMI (kg/m^2^) as categories, N (%)				
18.5–24.9, normal weight	20 (9.6)	11 (15)	9 (7)	0.18
25–29.9, overweight	74 (35.6)	25 (33)	49 (37)	
≥30, obese	114 (54.8)	39 (52)	75 (56)	

* *p*-values were obtained with Mann–Whitney U test for non-parametric numerical data. Abbreviations: M—male; F—female; DM—diabetes mellitus; AH—arterial hypertension; pSBP—peripheral systolic blood pressure; pDBP—peripheral diastolic blood pressure; PWV—pulse wave velocity; BBs—beta-blockers; ACEs—angiotensin-converting enzyme inhibitors; ARBs—angiotensin receptor blockers; Cas—calcium channel blockers; SMI—sarcopenic index; WHtR—waist-to-height ratio; WHR—waist to hip ratio; MUAC—middle upper arm circumference; WC—waist circumference; BMI—body mass index.

**Table 2:** Biochemical parameters regarding differences in phase-angle categories in all study participants.

	Total (*n* = 208)	Low Phase Angle (*n* = 75)	High Phase Angle (*n* = 133)	*p*-Value *
FBG (mmol/L), median (IQR)	7.4 (6.3–8.88)	7.45 (6.2–9.2)	7.3 (6.33–8.78)	0.87
HbA1c (%), median (IQR)	6.8 (6.3–7.7)	6.6 (6.3–7.2)	6.9 (6.3–7.9)	0.06
Alb (g/L), median (IQR)	43 (40–46)	42 (40–45.3)	43.1 (40–46)	0.65
Total cholesterol (mmol/L), median (IQR)	4.7 (4.1–5.75)	4.6 (4–5.3)	4.8 (4.1–5.8)	0.37
LDL (mmol/L), median (IQR)	2.7 (2.1–3.4)	2.6 (2.1–3.1)	2.8 (2.08–3.5)	0.18
HDL (mmol/L), median (IQR)	1.1 (0.9–1.3)	1.1 (0.9–1.3)	1.1 (0.9–1.3)	0.77
Tgl (mmol/L), median (IQR)	1.9 (1.4–2.5)	1.9 (1.6–2.36)	1.9 (1.4–2.68)	0.58
Uric acid (mmol/L), median (IQR)	414 (351–474)	419 (365.25–466.75)	405 (343–477)	0.60
E (×10^12^/L), mean (SD)	4.66 (4.24–5.03)	4.43 (4.02–5.03)	4.7 (4.33–5.04)	0.04
Hb (g/L), median (IQR)	136 (123–148)	128 (113–143)	139 (130–152)	<0.001
MCV (fL), median (IQR)	88.8 (85.9–91.93)	88.2 (85.1–91.1)	89 (86.3–92.8)	0.11
Creatinine (mmol/L), median (IQR)	114 (78.25–161.75)	129 (78–179)	110 (78–154)	0.23
Estimated GF using CKD-EPI (mL/min/1.73 m^2^), median (IQR)	53 (31–76.7)	40.93 (27.65–70.15)	57.2 (36.5–81.25)	0.02
ACR (mg/g), median (IQR)	2.89 (0.72–12.71)	1.69 (0.7–14.08)	3.15 (0.75–13.28)	0.69
Proteinuria (mg/24 h), median (IQR)	347 (97–1018)	425 (86–1447)	338.5 (99.75–944.5)	0.61
Albuminuria (mg/24 h), median (IQR)	97.5 (18.75–770)	149 (18–983)	76.5 (22.25–594.5)	0.56

* *p*-values were obtained with Mann–Whitney U test for non-parametric numerical data. Abbreviations: FBG—fasting blood glucose (mmol/L); HbA1c (%)—glycated hemoglobin; Alb—serum albumin (g/L); LDL—low-density lipoprotein cholesterol (mmol/L); HDL—high-density lipoprotein cholesterol (mmol/L); Tgl—triglycerides (mmol/L); E—erythrocyte count; Hb—hemoglobin (g/L); MCV—mean cellular volume (fL); GF—glomerular filtration; ACR—albumin to creatinine ratio (mg/g).

**Table 3:** Correlations between phase-angle and measured parameters (only statistically significant parameters shown).

	R	*p*-Value *
Age (years)	−0.308	<0.001
Body fat (%)	−0.184	0.01
Muscle mass (kg)	0.388	<0.001
Muscle mass (%)	0.194	0.01
Fat-free mass (kg)	0.393	<0.001
Skeletal muscle mass (kg)	0.504	<0.001
Skeletal muscle mass (%)	0.42	<0.001
SMI	0.346	<0.001
WC (cm)	0.147	0.04
Metabolic age (years)	−0.184	0.01
pDBP (mmHg)	0.224	0.01
E (×10^12^/L)	0.187	0.02
Hb (g/L)	0.31	<0.001
HbA1c (%)	0.16	0.04
Estimated GF using CKD-EPI (mL/min/1.73 m^2^)	0.169	0.02
PWV (m/s)	−0.192	0.04
Deli meats intake (frequency)	0.203	<0.001
Eggs intake (frequency)	0.147	0.03
Cheese intake (frequency)	0.147	0.03
Fruit intake (frequency)	−0.166	0.02
Red meat intake (MeDi adherence)	−0.141	0.04

* *p*-values were obtained with Spearman’s rank correlation. Abbreviations: SMI—sarcopenic index; WC—waist circumference; pDBP—peripheral diastolic blood pressure; E—erythrocyte count; Hb—hemoglobin (g/L); HbA1c—glycated hemoglobin; PWV—pulse wave velocity.

**Table 4:** Association between phase-angle and measured parameters adjusted for age, gender, and BMI.

	Adjusted for Age, Gender and BMI		
	Beta	*p*-Value	95% CI
Body fat (%)	−0.04	0.57	0.96 (0.85–1.09)
Fat-free mass (kg)	0.09	0.52	1.09 (0.83–1.44)
Muscle mass (kg)	−0.07	0.24	0.93 (0.83–1.05)
Skeletal muscle mass (kg)	0.16	<0.001	1.18 (1.09–1.27)
MUAC (cm)	0.18	0.03	1.20 (1.02–1.42)
HC (cm)	−0.04	0.27	0.96 (0.90–1.03)
pDBP (mmHg)	0.01	0.33	1.01 (0.99–1.04)
PWV (m/s)	−0.01	0.96	0.99 (0.63–1.56)
Hb (g/L)	0.03	0.01	1.03 (1.01–1.05)
HbA1c (%)	0.37	0.04	1.45 (1.01–2.08)
ACE inhibitors	0.48	0.15	1.62 (0.83–3.15)
Oral hypoglycemics	−0.81	0.08	0.45 (0.18–3.15)
Insulin	0.62	0.16	1.86 (0.79–4.40)
Red meat intake (frequency)	0.27	0.14	1.31 (0.92–1.86)
White meat intake (frequency)	0.46	0.06	1.59 (0.98–2.59)
Fast food intake (frequency)	0.38	0.10	1.46 (0.93–2.30)

Abbreviations: MUAC—middle upper arm circumference, HC—hip circumference, pDBP—peripheral diastolic blood pressure, PWV—pulse wave velocity, Hb—hemoglobin (g/L), HbA1c—glycated hemoglobin; ACE—angiotensin-converting enzyme, CI—confidence interval; OR—odds ratio.

## Data Availability

The raw data can be provided by the corresponding author via e-mail: josiparadic1973@gmail.com.

## Supplementary Materials

The following supporting information can be downloaded at: https://www.mdpi.com/article/10.3390/nu14235058/s1.
