# Supplementary material for: Phase Angle Association with Dietary Habits and Metabolic Syndrome in Diabetic Hypertensive Patients: A Cross-Sectional Study
Source: Nutrients. 2022 Nov 28;14(23):5058. doi: 10.3390/nu14235058 (PMC9738996; doi:10.3390/nu14235058)
Supplement: Supplementary file 1 [file nutrients-14-05058-s001.zip › nutrients-2048111-SI.pdf]

Table S1. Dietary habits of the total study population regarding differences in PhA

	Number (%) of participants			
	**Total (n=208)**	**Low Phase Angle (n=75)**	**High Phase Angle (n=133)**	***p*- Value***
Rapid weight changes	40 (19)	14 (19)	26 (20)	0.880
Last body weight measurement				
Do not remember	50 (24)	16 (21)	34 (26)	0.980
Yesterday	43 (21)	16 (21)	27 (20)	
One week ago	58 (28)	22 (29)	36 (27)	
One month ago	41 (20)	15 (20)	26 (20)	
Before more than one month	16 (7.7)	6 (8)	10 (7.5)	
Received any kind of dietary recommendation	109 (52)	37 (49.3)	72 (54.1)	0.510
Following dietary recommendations				
No	49 (45)	18 (48)	31 (43)	0.780
Yes	31 (28)	9 (24)	22 (31)	
Sometimes	29 (27)	10 (27)	19 (26)	
Regular stool	158 (76)	56 (75)	102 (77)	0.740
Presence of nausea	30 (14.4)	13 (17.3)	17 (12.8)	0.370
Loss of appetite	31 (14.9)	12 (16)	19 (14.3)	0.390
Alcohol consumption				
No	131 (63)	54 (72)	77 (58)	0.090
Yes	23 (11)	8 (11)	15 (11)	
Sometimes	54 (26)	13 (17)	41 (31)	
Physical activity	108 (51.9)	37 (49.3)	71 (53.4)	0.720
Number of meals				
1 – 2	17 (8)	5 (7)	12 (9)	0.830
2 – 4	162 (78)	59 (79)	103 (77)	
>4	29 (14)	11 (15)	18 (14)	
Meal preparation				
Personally	64 (50)	21 (53)	43 (48)	0.660
Family member	65 (50)	19 (48)	46 (52)	
Eating breakfast	178 (85.6)	68 (90.7)	110 (82.7)	0.120
Eating snacks	120 (57.7)	41 (54.7)	79 (59.4)	0.510
Adding salt to meals				
No	57 (27)	22 (29)	35 (26)	
Yes	84 (40)	33 (44)	51 (38)	0.440
Sometimes	67 (32)	20 (27)	47 (35)	
Prescribed with ONS	26 (12.6)	13 (17.3)	13 (9.9)	0.130
**Food Frequency Questionnaire (FFQ)**				
Red meat	2 (2 - 3)	2,5 (2 - 3)	3 (2 - 3)	0,003
White meat	3 (3 - 3)	2 (2 - 3)	3 (3 - 3)	0,02
Fish	2 (2 - 2)	2 (2 - 2)	2 (2 - 3)	0,75
Deli meats	2 (1 - 3)	2 (1 - 3)	2 (1 - 3)	0,09
Eggs	2 (1 - 3)	2 (1 - 3)	2 (2 - 3)	0,07
Milk	3 (1 - 4)	3 (1 - 4)	3 (1 - 4)	0,55
Yoghurt	3 (1,25 - 4)	3 (1 - 4)	3 (1,5 - 4)	0,84
Heavy cream	0 (0 - 1)	0 (0 - 1)	0 (0 - 1)	0,71
Cheese	3 (3 - 4)	3 (3 - 4)	3 (3 - 4)	0,07
Fruit	4 (3 - 4)	4 (4 - 4)	4 (3 - 4)	0,15
Vegetables	4 (3 - 4)	4 (3 - 4)	4 (3 - 4)	0,92

Dried fruit	0 (0 - 1)	0 (0 - 1)	0 (0 - 1)	0,98
Nuts	1 (0 - 3)	1 (0 - 3)	1 (1 - 2)	0,54
Seeds	0 (0 - 1)	0 (0 - 1)	0 (0 - 1)	0,59
Legumes	2 (1 - 2)	2 (1 - 2)	2 (1 - 2)	0,64
Bread	4 (4 - 4)	4 (4 - 4)	4 (4 - 4)	0,62
Pasta	2 (1 - 2)	2 (1 - 2)	2 (1 - 2)	0,25
Cornflakes, muesli	0 (0 - 0)	0 (0 - 0)	0 (0 - 0)	0,88
Oat flakes	0 (0 - 1)	0 (0 - 1)	0 (0 - 1)	0,80
Rice	2 (1 - 2)	2 (1 - 2)	2 (1 - 2)	0,74
Other grains (quinoa, amaranth, millet…)	0 (0 - 1)	0 (0 - 1)	0 (0 - 1)	0,40
Fast food	0 (0 - 1)	0,5 (0 - 1)	1 (0 - 1)	0,003
Sweets	2 (1 - 3)	1 (1 - 3)	2 (1 - 3)	0,11

*0- never, 1- rare, 2- once a week, 3- twice or thrice a week, 4-once a day
